# Microbial contamination of multiple-dose preservative-free hospital ophthalmic preparations in a tertiary care hospital

**DOI:** 10.1016/j.aopr.2022.100046

**Published:** 2022-03-30

**Authors:** Somporn Chantra, Pinyada Hathaisaard, Andrzej Grzybowski, Paisan Ruamviboonsuk

**Affiliations:** a Department of Ophthalmology, Rajavithi Hospital, College of Medicine, Rangsit University, Thailand; b University of Warmia and Mazury, Olsztyn, Poland; c Institute for Research in Ophthalmology, Poznan, Poland

**Keywords:** Microbial contamination, Multiple-dose eye drops, Anti-infective eye drops, Preservative-free, Hospital ophthalmic preparation

## Abstract

**Background:**

It is possible that preservative-free eye drops can be contaminated. The aim of this study was to assess the incidence of microbial contamination of preservative-free hospital-prepared anti-infective eye drops and investigate factors that contribute to contamination. This finding may help to raise awareness of this problem to medical healthcare staff and patients in order to prevent the transmission of microorganisms from eye drops to the patients through treatment of pre-existing eye diseases.

**Methods:**

Two hundred and ninety-five eye drop bottles were collected from patients attending Rajavithi Hospital Ophthalmologic outpatient and inpatient department, including both those used by patients at home and those administered in the hospital by medical staff. Samples were taken from the tips of droppers and bottles, and the residual fluid inside the bottles was then cultivated onto different culture plates. The culture results were identified and analyzed according to various factors related to both individual users and the bottles.

**Results:**

Seven different types of eye drops were collected and 71 (24.06%) of the 295 bottles were contaminated. Vancomycin eye drops were the most contaminated. Twenty-six different types of pathogens were identified, most frequently mold (42.98%), and the amount of contamination was higher in tips than in residual fluid inside the bottle. There was no statistically significant difference in contamination between patients used eye drops collected in outpatient units (32.14%) and medical staff used eye drops collected in inpatient settings (23.22%). The only factor that was statistically significant was the number of eye drops used per person. We found that samples from patients who used only up to 2 eye drops suffered contamination (42.8%) more than those from their counterparts who used at least 3 (22.18%), *P* ​= ​0.02.

**Conclusions:**

Of these preservative-free hospital preparations anti-infective eye drops, 24.06% were contaminated. The number of eye drops used per person was statistically significant in triggering contamination. There is a possibility of number of eyedrops use person may trigger contamination.

## 1 Introduction

Many different types of eye drops, both single- and multiple-dose units, are used in hospitals, some of which are manufactured by commercial companies and others that are prepared in the hospital. Since some commercially produced eye drops are unavailable to patients because of their frequency of use, drug stability, or cost, yet they are essential for treatment. In such cases, hospital-made preservative-free eye drops can be supplied by the hospital's in-house pharmacy. It is well known that without preservatives, ophthalmic solutions may become contaminated.^1^ It is possible that microbial contamination may alter the pH of preparations and reduce the eye drops' efficacy.^2^^,^^3^ Therefore, maintaining sterility during eye drop use is a crucial measure.

In two reports of contamination of preservative-free hospital-prepared eye drops, 8.4%^4^ and 16.7%^1^ were found to be contaminated, and the bottles used by patients at home were more tainted than those used by medical staff in inpatient settings.^4^^,^^5^ Another study showed there was no difference between contamination rates in eye drops administered by staff in outpatient departments and those instilled in wards using shared medications in multi-user settings; however, inter-patient transmission of microorganisms could have influenced the results.^2^ Prior studies have revealed that the specific area of contamination was considerable; generally, the tip of the bottle was more likely to be contaminated than the residue inside.^2^^,^^5^, ^6^, ^7^, ^8^, ^9^, ^10^ No evidence found of increased contamination in plastic containers over glass bottles in eye drops containing preservative.^11^

The preservative-free eye drops supplied by Rajavithi Hospital are stored locally in 2 types of containers: plastic and amber glass. The plastic containers, with a sealed cap, are designed for multiple-dose use, while the glass containers are used for droppers. Some eye-drop patients have to prepare their own solution at home from powder, and they need to refrigerate the preparation to keep it at a cool temperature in order to inhibit the growth of microorganisms.

This study was conducted at the Department of Ophthalmology of Rajavithi Hospital, Thailand. The primary outcome was to study the contamination rate of multiple-use, preservative-free, hospital-produced eye drops used both by patients at home and by medical professionals in inpatient settings. The secondary outcome was to analyze the pattern of bacterial and fungal contamination in terms of type of medication, type of bottles, location of sample collected, duration and frequency of use, and patient variables to reveal the risk factors which may contribute to contamination. To identifying the risk factors for contamination, may help to raise awareness of the problem and develop rules or guidelines for medical healthcare staff and patients to prevent the transmission of microorganisms from eye drops to the patients through treatment of pre-existing eye diseases.

## 2 Material and methods

The protocol of this research was reviewed and approved by the ethics committee of Rajavithi Hospital (No. 78/2562). This was a cross-sectional study performed in the Department of Ophthalmology, Rajavithi Hospital, from March 2019 to July 2019, in which eye drops were collected from the inpatient and outpatient departments. The inclusion criteria were patients, regardless of diagnosis, who used topical anti-infective medications, preservative-free and multiple-use eye drops only, prepared by Rajavithi Hospital. The hospital-made preservative-free eye drops included: antibiotics Amikacin 50 ​mg/ml, Ceftazidime 50 ​mg/ml, Cefazolin 50 ​mg/ml, Vancomycin 50 ​mg/ml; antifungals Amphoterincin B 1.7 ​mg/ml, Natamycin 50 ​mg/ml, Voriconazole 10 ​mg/ml. Bottles used for fewer than 2 days or which had passed their expiry date were excluded. Samples of each preservative-free eye drop bottle were tested for sterility, so that contamination of the unopened bottles was highly unlikely. We classified the data into 2 groups. Group 1 consisted of hospital-used preparations such as eye drops instilled by nurses or ophthalmologists, and samples collected from the inpatient department. In the case of fortified eye drops, preparation was performed by the pharmacist or ward nurse under aseptic conditions. Group 2 comprised eye drops used at home by patients or others (usually relatives) at home, and the samples were collected at the outpatient clinic at scheduled follow-up visits. The patients were asked to bring eye drops to the outpatient clinic after using them for more than 2 days. All patients gave informed consent for their used eye drops to be analyzed, and all samples were discarded after analysis.

Patient factors were collected for the two groups, including age, sex, level of education, eye condition that required the studied eye drops, best corrected visual acuity (VA) in better eye, presence of systemic disease, whether the drops were self-administered (and if not, details of the helper were recorded). Secondly, details of eye-drop factors were collected including type, storage method, duration from the first opening of the eye drop bottles to sampling, self-report of contamination between eye drop tips/droppers and eyelids/eyelashes, frequency of use, and design of containers. Visual acuity was measured by Snellen chart and then converted into LogMAR values for analysis.

### 2.1 Sample collection and microbiological analysis

All fortified antimicrobials eye drops in the study were prepared from a hospital pharmacy that follows the United States Pharmacopeial Convention (USP) <797>.^12^ The USP<797> provides practice and quality standards for all personnel who have a responsibility in the pharmacy compounded sterile preparations (CSPs). Sterility testing was performed according to <71>Sterile tests, which is a protocol under USP<797>, using the membrane filtration method. We use membrane filters with pore sizes not greater than 0.45 μm to retain microorganisms and remove antimicrobial agents. We then transfer the whole membrane to the culture medium and incubate the media for at least 14 days. Furthermore, before beginning the study, we randomized the eye drops preparations that haven't been used to be cultured for confirmation that the preparations are culture-negative.

Due to contamination issues, preservative-free eye drops available commercially are generally discarded after a single use. However, preservative-free eye drops from CSPs can be discarded according to beyond-use dates (BUDs).^13^ This is recommended by USP<797>. USP<797> defines BUDs as the date or time after which CSPs may not be stored or transported and is calculated from the date or time of compounding.

In our study, all fortified antimicrobial eye drops were CSPs prepared by the hospital pharmacy and discarded by BUDs based on the recommendation by USP<797>. USP<797> limits BUDs of CSPs based on categories. Our hospital-made preservative-free eye drops can be classified into category 2; according to which, they can be used for 4 days at controlled room temperature or for 10 days refrigerated from the time of opening. We have certainly complied with the BUDs recommended by USP<797>.

A total of 295 eye drop containers were collected from Rajavithi Hospital Ophthalmology Department, 267 from inpatient departments and 28 from outpatient departments. The sources of microbial analysis were from 2 sites: the tip of the droppers or the tip of the eye-drop bottles and residual eye drops in the bottles. We collected the samples in accordance with standard procedure, sterile saline was used to moisten the sterile cotton swab, which was then used to wipe the tip of the droppers or the tip of the eye-drop bottles after which the sample was inoculated on culture plates including chocolate agar, blood agar and sabouraud dextrose agar. The residual eye drops in the bottles were drawn off using a needle and syringe to avoid contamination at the tip of the eye drop and then dropped on each of the media as from the tip of the bottles, a single drop per sample. As in other studies, samples collected from the eye drops that have already been used were directly inoculated on the media without removing antimicrobial or antifungal substances in microbial analysis. A flame-sterilized loop which was allowed to cool was used to streak the drops evenly on the agar surface using the spread plate technique to isolate and count the pathogens. When the media were completely inoculated, chocolate agar and blood agar were incubated at 35^๐^c routinely for 24 ​h and examined daily for pathogen growth. Re-incubation for an extra 48 ​h was indicated when no pathogen growth was detected. Sabouraud agar were incubated at 25^๐^c, and if yeasts or fungi were detected, the media were incubated for up to one month. All organism growths from culture media were considered critical and identified. For the culture of media in which colonies were found. The microorganisms were identified by matrix-assisted laser desorption ionization-time of flight mass spectrometry (MALDI-TOF MS) methods. The MALDI-TOF MS was done on a Microflex LT instrument (Bruker Daltonics, Bremen, Germany) using the FlexControl 3.4 software (Bruker Daltonics, reference library version 4.0.0.1 software, containing 5627 species).^14^ The MALDI-TOF MS is used to identify microbes by comparing the peptide mass fingerprint (PMF) of unknown organisms to the PMFs in the commercially provided database.

The MALDI-TOF MS has a limitation in diagnostic mycology, particularly for molds identification, due to the lack of fungal reference spectrum in commercial databases and the requirement for extended sample preparation.^15^ From our results, as molds were detected, species identification was therefore limited.

### 2.2 Statistical analysis

In this cross-sectional study, the sample size was calculated with the presumption of a 16.7%^1^ contamination rate of multi-dose preservative-free ophthalmic drops based on a preparation study in Japan. The estimate proportion formula was used to calculate the subjects with a confidence interval of 95%. The margin of error was estimated based on 20% of the previous presumption. The calculated sample size(n) for the chosen parameters was therefore 505.

$$n=\frac{Z_{\text{α/}2}^{2}p\left(1-p\right)}{d^{2}}$$

The parameters were as follows:

n ​= ​calculated sample size
Z _α/2_ ​= ​standard score corresponding to 95% confidence level, then Z _α/2_ ​= ​1.96
p ​= ​0.16 (estimated prevalence, taken from Atsuyuki Saisya study, 2016)
d ​= ​margin of error 20% of prevalence (0.2 ​× ​0.16 ​= ​0.032)
$n=\frac{1.96^{2}\times 0.16\times (1-0.16)}{{\left(0.032\right)}^{2}}=505$

The calculated sample size(n) for the chosen parameters was therefore 505. The number of sample sizes that can be collected in the study did not reach the number calculated from sample size calculation due limitation of time for data collection. The samples from the outpatient department were lower than designated due to the compliance and co-operation of the patients; therefore, this was a limitation, as our samples from home use were far fewer than those from hospital use.

The data were presented as numbers and percentages or mean and standard deviation. Statistical analysis was performed using SPSS for Windows Software (Version 17.0, SPSS, Inc.). Chi-square pair *t*-test, Fisher exact test and independent *t*-test (for continuous variables) were used to test for statistical significance of differences in incidence of contamination in eye drops between all groups. Differences were considered statistically significant when the *P*-value was less than 0.05.

## 3 Results

A total of 295 eye drop bottles were recruited from 285 patients: 267 bottles from 264 patients in group 1 (medical professionals-used) and 28 bottles from 21 patients in group 2 (patients-used). The demographic information of the two groups is summarized in Table 1. There were 129 males and 156 females. Median best-corrected visual acuity of better eye was 20/20 range between 20/20 to light perception. The ophthalmic conditions that required the sample eye drops were mostly infectious keratitis (95.43%, 272/285) followed by post-intraocular surgery (4.21%, 12/285 bottles), and lastly corneal edema (0.35%, 1/285). Subjects’ level of education was classified into 2 groups: up to primary (4.21%, 12/285); at least secondary (95.78%, 273/285). The presence of underlying systemic disease was recorded, including hypertension, diabetes, dyslipidemia and heart disease. The majority of the patients (55.08%, 157/285) had no underlying disease. Table 2 shows the type of medications, number of bottles collected (group1/group2), number of bottles collected and contaminated bottles categorized by site of contaminated found: amikacin (6/0), ceftazidime (72/9), cefazolin (32/0), vancomycin (70/11), amphotericin B (77/4), natamycin (8/3), voriconazole (2/1). Seventy-one (24.07%) of 295 bottles had pathogens detected on culture media. The medications were grouped into two categories: antibiotics and antifungals. In the antibiotic group, contamination rates with amikacin, ceftazidime, cefazolin and vancomycin were 16.7% (1/6 bottles), 19.75% (16/81 bottles), 15.63% (5/32 bottles) and 29.6% (24/81 bottles) respectively. With regard to the antifungal group, contamination rates of amphotericin B, natamycin and voriconazole were 25.93% (21/81 bottles), 18.18% (2/11 bottles) and 66.7% (2/3 bottles), respectively. The contamination rates in each group were 23% (46/200) in the antibiotics group, 26.31% (25/95) in the antifungals group. No significantly higher contamination was found in the antifungals group compared to antibiotics groups (*P*= 0.53).

**Table 1:** Demographic data.

Variables	Group 1	Group2	Total	*P*
Number of bottles collected	267	28	295	
Number of patients	264	21	285	
Age (years)				
Mean ​± ​SD	58.70 ​± ​14.51	55.24 ​± ​14.55		0.293
**Sex (%)**				0.11
Female	148 (94.87%)	8 (5.13%)	156	
Male	116 (89.92%)	13 (10.08%)	129	
BCVA of better eye LogMAR (median)	0.0 (0–1)	0.3 (0.0–2.7)		<0.001∗
**Eye disease for eye drops (%)**				<0.001∗
Infectious keratitis	257 (94.49%)	15 (5.51%)	272	
Post intraocular surgery	7 (58.33%)	5 (41.67%)	12	
Corneal edema	0	1 (100%)	1	
Level of education (%)				<0.001∗
Up to primary	0	12 (100%)	12	
At least secondary	264 (96.70%)	9 (3.30%)	273	
Underlying systemic disease (%)				0.268
Yes	143 (91.08%)	14 (8.92%)	157	
No	121 (94.53%)	7 (5.47%)	128	

Group 1 ​= ​medical professionals-used, Group 2 ​= ​patients -used.

∗Significant *P*–value ​< ​0.05.

**Table 2:** Type of medications, number of bottles collected and contaminated bottles categorized by site of contaminated found.

Type of medications	Number of bottles collected			Number of contaminated bottles categorized by site of contaminated found			
	Group 1	Group2	Total	Tip only	Residual fluid in the bottle only	Both tip and residual fluid in the bottle	Total (%)
**Antibiotic**							
Amikacin	6	0	6	1	0	0	1 (16.7%)
Ceftazidime	72	9	81	10	3	3	16 (19.75%)
Cefazolin	32	0	32	4	0	1	5 (15.63%)
Vancomycin	70	11	81	10	7	7	24 (29.63%)
			**200**				**46(23.0%)**
**Antifungal**							
Amphotericin B	77	4	81	10	7	4	21 (25.93%)
Natamycin	8	3	11	0	0	2	2 (18.18%)
Voriconazole	2	1	3	0	2	0	2 (66.70%)
			**95**				**25(26.31%)**
							
**Total**			**295**				**71(24.07%)**

Group 1 ​= ​medical professionals-used, Group 2 ​= ​patients-used.

Table 3 shows that of the 71 contaminated bottles, 35 (49.2%, 35/71) were contaminated only at the tip of bottles or droppers, 19 (26.7%, 19/71) had contamination only in the residual fluid, and 17 (23.9%, 17/71) showed contamination of both tips and residual fluid. Statistical significance was found in tip contamination in the medical professionals-used group (*P*<0.001) while in the patients-used group, statistically significant contamination was found in both tips and residual fluid (*P*<0.001). The identities of the microorganisms found in eye drop samples are presented in Table 4; it should be noted that in some samples more than one microorganism was found. Most of the detected microorganisms were fungus with a percentage of 50. Molds (filamentous fungi; Aspergillus, Fusarium) were predominant with 42.98% occurrence. There was also a wide range of Gram-positive bacteria; in particular, Micrococcus Luteus and Staphylococcus Capitis, which occurred in 7.98% and 7.02% respectively, were part of the normal skin or conjunctiva flora. Other bacteria and fungi were also human flora or environmental flora that can be airborne or contaminated from soil. Table 5 shows the factors related to contamination. The contamination rate in Group 1 (medical professionals-used) of 23.22% was lower than that of Group 2 (patients-used) of 32.14% but not statistically significant. Other non-statistically significant factor for contamination were gender, age, visual acuity of better eye, education level, underlying systemic disease, the condition causing the need for eye drops, self-report of contamination between eye drop tips/droppers and eyelids/eyelashes, self-used or other-used, storage method, duration of use, frequency of use, and design of containers. The only factor that was statistically significant was the number of eye drops used per person. We found that samples from patients who used only up to 2 eye drops suffered contamination (12/29, 41.38%) more than those from their counterparts who used at least 3 (59/266, 22.18% *P* = 0.02) (see Table 6).

**Table 3:** Number of contaminated found categorized by site of contamination and group of patients.

Site of contaminated found	Number of contaminated bottles		
	Group 1	Group2	Total (%)
**Tip only**	34	1	35 (49.2%)
**Fluid in the bottle only**	17	2	19 (26.7%)
**Both tip and residual fluid in the bottle**	11	6	17 (23.9%)
			**71 (100%)**

Group 1 ​= ​medical professionals-used, Group 2 ​= ​patients-used.

**Table 4:** List of pathogens found in contaminated sample.

Pathogens	Number of contaminated samples			Percentage of occurrence
	Group 1	Group2	Total	
**Mold**	41	8	49	42.98%
**Micrococcus Luteus**	5	4	9	7.89%
**Staphylococcus Capitis**	8	0	8	7.02%
**Bacillus Cereus**	3	3	6	5.26%
**Acinetobacter**	1	3	4	3.51%
**Staphylococcus Epidermidis**	3	1	4	3.51%
**Candida**	1	2	3	2.63%
**Kocuria Rhizophila**	2	1	3	2.63%
**Staphylococcus Hominis**	3	0	3	2.63%
**Staphylococcus Hemolyticus**	3	0	3	2.63%
**Staphylococcus Saprophyticus**	3	0	3	2.63%
**Yeast not Candida**	1	2	3	2.63%
**Pseudomonas Aeruginosa**	0	2	2	1.75%
**Trichosporon asahii**	0	2	2	1.75%
**Arthrobacter**	1	1	1	0.88%
**Bravibactrium Casei**	1	1	1	0.88%
**Corynebacterium**	1	0	1	0.88%
**Escherichia Coli**	1	0	1	0.88%
**Exiguobacterium**	1	0	1	0.88%
**Neiserria**	1	0	1	0.88%
**Rothia**	1	0	1	0.88%
**Solibacillus**	1	0	1	0.88%
**Staphylococcus Aureus**	1	0	1	0.88%
**Staphylococcus Lugdunensis**	0	1	1	0.88%
**Staphylococcus Sciuri**	1	0	1	0.88%
**Stenoropnomonas Maltophilia**	1	0	1	0.88%
	**85**	**29**	**114**	

Group 1 ​= ​medical-professionals- used, Group 2 ​= ​patients-used.

**Table 5:** Factors related to contamination.

Factors	Contaminated	Non-contaminated	*P*
**Group, number of bottles (%)**			0.293
Group 1	62 (23.22%)	205 (76.78%)	
Group2	9 (32.14%)	19 (67.86%)	
**Gender number of bottles (%)**			0.305
Female	35 (21.74%)	126 (78.26%)	
Male	36 (26.87%)	98 (73.13%)	
**Age, years (mean ​+ ​SD)**	55.99 ​± ​15.52	58.58 ​± ​14.31	0.194
**Mean visual acuity, LogMAR (mean ​+ ​SD)**	0.06 ​± ​0.33	0.04 ​± ​0.26	0.567
**Education level, number of bottles (%)**			0.686
Up to primary	4 (28.57%)	10 (71.43%)	
At least secondary	67 (23.84%)	214 (76.16%)	
**Underlying disease, number of bottles (%)**			0.327
No	44 (26.19%)	124 (73.81%)	
Yes	27 (21.26%)	100 (78.74%)	
**Eye disease for eye drops, number of bottles (%)**			0.504
Infectious keratitis	70 (24.05%)	221 (75.95%)	
Corneal edema	1 (50%)	1 (50%)	
Post intraocular surgery	0	2 (100%)	
**Report of usage contamination by user, number of bottles (%)**			0.715
Never	65 (23.81%)	208 (76.19%)	
Sometimes	6 (27.27%)	16 (72.73%)	
**User, number of bottles (%)**			0.911
Self-used	6 (25.0%)	18 (75.0%)	
Other-used	65 (23.99%)	206 (76.01%)	
**Storage, number of bottles (%)**			0.424
In refrigerator	70 (23.89%)	223 (76.11%)	
Out of refrigerator	1 (50%)	1 (50%)	
**Mean duration of usage, days (mean** ​± ​**SD)**	3.23 ​± ​1.07	3.16 ​± ​1.23	0.671
**Container, number of bottles (%)**			0.145
Glass container	69 (23.63%)	223 (76.37%)	
Plastic container	2 (66.67%)	1 (33.33%)	
**Number of eye drops use per person, number of bottles (%)**			0.022∗
Up to 2 bottles	12 (41.38%)	17 (58.62%)	
>2 bottles	59 (22.18%)	207 (77.82%)	

Group 1 ​= ​medical professionals-used, Group 2 ​= ​patients-used.

∗Significant *P*–value ​< ​0.05.

**Table 6:** Factors related to molds contamination.

Factors	Mold-contaminated	Not-mold contaminated	*p*
**Group, number of bottles (%)**			0.541
Group 1	31 (11.61)	31 (11.61)	
Group2	4 (14.29)	5 (17.86)	
**Gender number of bottles (%)**			0.494
Female	16 (9.94)	19 (11.80)	
Male	19 (14.18)	17 (12.69)	
**Age, years, mean ​+ ​SD**	56.74 ​± ​13.59	55.25 ​± ​17.34	0.393
**Mean visual acuity, LogMAR, mean ​+ ​SD**	0.103 ​± ​0.46	0.02 ​± ​0.07	0.361
**Education level, number of bottles (%)**			0.921
Up to primary	2 (14.29)	2 (14.29)	
At least secondary	33 (11.74)	34 (12.10)	
**Underlying disease, number of bottles (%)**			0.445
No	20 (11.90)	24 (14.29)	
Yes	15 (11.81)	12 (9.45)	
**Eye disease for eye drops, number of bottles (%)**			0.475
Infectious keratitis	34 (11.68)	36 (12.37)	
Corneal edema	1 (50)	0	
Post intraocular surgery	0	0	
**Report of usage contamination by user, number of bottles (%)**			0.196
Never	34 (12.45)	31 (11.36)	
Sometimes	1 (4.55)	5 (22.73)	
**User, number of bottles (%)**			0.993
Self-used	3 (12.50)	3 (12.5)	
Other-used	32 (11.81)	33 (12.18)	
**Storage, number of bottles (%)**			0.235
In refrigerator	34 (11.60)	36 (12.29)	
Out of refrigerator	1 (50)	0	
**Mean duration of usage, days, mean** ​± ​**SD**	3.14 ​± ​0.94	18.17 ​± ​6.97	0.048∗
**Container, number of bottles (%)**			0.015∗
Glass container	35 (11.99)	34 (11.64)	
Plastic container	0	2 (66.67)	
**Number of eye drops use per person, number of bottles (%)**			0.022∗
Up to 2 bottles	4 (13.79)	8 (27.59)	
>2 bottles	31 (11.65)	28 (10.53)	

Group 1 ​= ​medical professionals-used, Group 2 ​= ​patients-used.

∗Significant *P-*value ​< ​0.05.

## 4 Discussion

There have been few studies reporting contamination rates of preservative-free ophthalmic solution, and they found a range from 2.0%^16^ to 16.7%.^1^ In this study, the contamination of preservative-free hospital-made anti-infective eye drops were 24.07%, which was higher than those of previous studies. Eye drops can be contaminated by the dropper, the cap, or the tip of the container with hands, eyelids, lashes or facial skin, which support the organisms detected mainly from skin flora and the environment^2^^,^^5^ We found that the tip of the bottle or dropper was most contaminated, and this was similar to the results of earlier studies.^2^^,^^5^, ^6^, ^7^, ^8^, ^9^, ^10^

In particular, in a glass bottle with a dropper attached to the cap, fluid in the bottle is exposed to the outside environment when the pipette is pulled out during administration. The tip of a dropper which could be contaminated from skin or eyelashes may become immersed in the residual fluid in the bottle; therefore, it is possible that discarding a dropper more frequently before the medication has expired may result in less contamination. One study suggested that releasing a single drop from preparation prior to instillation could reduce the risk of inoculating the organisms at the tip of dropper or bottle into the patient's eyes.^10^

We found that molds (Aspergillus or Fusarium) were the predominant contaminant in eye drops, followed by Gram-positive bacteria, and this differs from the findings of some other studies^2^^,^^5^, ^6^, ^7^, ^8^^,^^10^^,^^17^, ^18^, ^19^ which reported that Gram-positive bacteria were the most common contaminants. However, a study from Japan^1^ reported Candida as the predominant organism, and another study from Thailand^20^ found Aspergillus was the most common contaminating organism from Autologous serum tear. As mentioned above, we thought that the organism cultured from eye drops may be a result of the tropical climate of the area, especially Southeast Asia, where fungals are the predominant organisms.

Although antifungal medications had the smallest incidence of fungal contamination compared with the other medication groups due to its antifungal property, it should be noted that fungal organisms could still contaminate in spite of antifungal medication.

We found that fungal contamination was more common in antibiotics than antifungals eye drops which may be explained by the absence of antifungal properties in antibiotics eye drops. Still, this finding raises concern about fungal contamination of preservative free antibiotic eye drops. There is a marketed formulation of moxifloxacin 0.5% ophthalmic solution [Vigamox® (Alcon, Fort Worth, TX)] which has no preservative. It was approved by the FDA for treating bacterial conjunctivitis.^21^ Mack and colleagues found fungal contamination in 13 from 32 (41%) bottles of moxifloxacin used by 32 infectious keratitis patients.^22^ However, those positive cultures were obtained from the exterior and interior surfaces of the bottles. It was inconclusive that fungal contamination of moxifloxacin bottles was the cause of fungal keratitis in some patients in the study.

The normal flora of skin and conjunctiva in humans contain numbers of bacteria, most of which are Gram positive while Gram-negative bacteria constitute a smaller proportion; however, dust particles or other materials may get trapped under the nail, depending on what the nail is in contact with. Fungi which can be found under the nail are mostly *Aspergillus*, *Penicillium*, *Cladosporium*, and *Mucor*.^23^ Personal hygiene, therefore, with special attention to nail hygiene with proper hand washing before drug administration, should be emphasized to every eye drop user, especially in countries where agriculture is the main occupation.

Even though bacterial normal flora are described as being commensal and not harmful to humans, and also part of our protective mechanism, most of them are either pathogens or opportunistic pathogens, and some of them may be considered as major pathogens in particular situations in humans such as *Staphylococcus aureus, Neisseria meningitidis,* Propionibacterium, Corynebacterium, *Pseudomonas aeruginosa*, Haemophilus influenzae *Escherichia coli, and Pseudomonas aeruginosa*.^24^ Moreover, some of the commensal bacteria of the conjunctiva, when introduced to the intraocular space might lead to serious infections, including endophthalmitis. One of the most common bacteria found on the surface of the eye is coagulase-negative staphylococci (CoNS).^25^ These are assumed to be commensal bacteria, colonizing the mucosa and lid margins. CoNS are the most commonly found bacteria, detected in up to 100% of positive conjunctival cultures taken from patients preoperatively, with *Staphylococcus epidermis* the predominant species.^25^ It is well-known that the major source of infection leading to post-operative endophthalmitis is the patient's own ocular surface flora with CoNS as the leading sort of bacteria.^26^ Unfortunately, those were all found in samples in our study, and since patients who need preservative-free hospital-made eye drops usually have a compromised corneal surface, care should be taken to avoid contamination of the solutions.

Our study found that no significant difference in contamination between patients-use and medical professional-use setting, and this is in keeping with the results of other study.^2^ Other studies, in contrast, have found difference in contamination in two setting.^4^^,^^5^ One study observed patients on video recordings while they self-administered their eye drops and found that patients who were inexperienced with eye drop use showed poor instillation techniques, failing to wash hands and inadvertently contaminating bottle tips.^27^ Training of patients to be experienced and aware of hygiene practices in drop instillation techniques are now being in hospital discharge planning is most probably the reason for our finding. We did not find any statistically significant correlation with other factors such as gender, age, visual acuity of better eye, education level, underlying systemic disease, the condition causing the need for eye drops, self-report of contamination between eye drop tips/droppers and eyelids/eyelashes, self-used or other-used, storage method, duration of use, frequency of use, and design of containers We found that all antibacterial, amphotericin B and natamycin medications are all kept in glass containers, except voriconazole is housed in plastic bottle, and this may have confounded the results of the study. Clearly, future research is needed to clarify this issue. The only factor that was statistically significant was the number of eye drops used per person. The samples from patients who used only up to 2 eye drops suffered contamination more than those from their counterparts who used at least 3. This finding may explain by patients extra careful when using a greater number of drops used per person.

Interestingly, in the subgroup contaminated with mold, total 35 bottles collected from 16 patients. Only 4 patients were found fungal positive from corneal scrape culture with diagnosis of fungal keratitis; 3 infectious keratitis patients showed no organism from corneal culture; 3 patients were found to have bacterial pathogens from corneal scrape with diagnosis of bacterial keratitis; 6 patients were used the study eye drops for surgical prophylaxis after intraocular surgery. All cases of infectious keratitis in this subgroup were healed with corneal scarring. Twelve patients with non-fungal keratitis and other diagnosis in this subgroup, none of them were present with secondary fungal infection after preservative-free hospital made eye drops were used and no antifungal eye drops needed. From our finding it has not been demonstrated secondary ocular fungal infection from fungal-contaminated preservative-free hospital made eye drops. Although there is a possibility that contaminated may occur in preservative free hospital made eye drops, but it still desirable in certain conditions such as severe infectious keratitis. The American Academy of Ophthalmology (AAO) 2018 Preferred Practice Pattern recommended fortified antibiotics or fourth-generation fluoroquinolones for treating bacterial keratitis.^28^ However, in cases of severe bacterial keratitis, fortified antibiotics which are non-preserved are still preferable in current practice. As we know, there was no evidence of secondary infection from contaminated hospital made eye drops, so we conclude that preservative free hospital made eye drop still can be used with caution and awareness of contamination.

Microorganisms can be probably introduced into eye drops during manufacturing, storage, or normal patient use. To assess the adequacy of antimicrobial properties of the eye drops either from the preservatives system or the properties of products themselves in a multi-dose or unit doses formulation, The Antimicrobial Effectiveness Test (AET) also known as Preservative efficacy tests (PETs) is utilized.^29^ AET is performed to ensure that antimicrobial effects are sufficient to inhibit or kill the microorganisms in the formulation for the entire shelf-life or during the use of the products. It is a microbial challenge method, often included in the development phase and stability testing protocols. The test entails inoculating a predetermined number of microorganisms.

The effectiveness of the antimicrobial properties is assessed by comparing the initial level of microorganisms in the test sample over a 28-day period to the acceptance criteria outlined in the compendial guidance documents.

The guidance documents for AET are the United States Pharmacopeia (USP) <51> Antimicrobial Effectiveness Testing, the European Pharmacopeia (EP) 5.1.3 Efficacy of Antimicrobial Preservation, and the Japanese Pharmacopeia (JP) 19, Preservative Effectiveness Tests. The comparison of each AET was previously described.^29^

It should be noted that there a possibility that microorganisms will contaminate eye drop products even though the preservatives are added to provide some antimicrobial activity. The incidence of microbial contamination of eyedrops with preservatives was varied ranging from 0.07% to 96.46%,^5^^,^^6^^,^^18^^,^^30^, ^31^, ^32^ which was found in artificial tear, antiglaucoma, fluorescein, anesthetic, atropine, cyclopentolate, tropicamide, and phenylephrine topical medications. Some of which are drugs that usually be used chronically for treating chronic diseases, some are used for diagnostic in the clinic. Therefore, the contamination of these multi-dose eye drops, although they contain preservatives, should also be considered.

## 5 Limitations

There were several limitations to this study. First, the numbers of samples from inpatient and outpatient units were very different, with the samples from outpatient departments less than designated due to the compliance and co-operation of the patients. Secondly, plastic containers were used to contain voriconazole, while glass containers were mostly used for others anti-infective agents, and this may have affected contamination rates. There were only samples with preservatives-free solutions tested and we did not compare them to the solutions containing preservatives.

Finally, the relatively small number of some eye drops, such as voriconazole, might have affected percentages of contamination due to the ratio of positive cultures to total amounts of eye drops.

## 6 Conclusions

Of the 295 eye drop containers recruited from 285 patients, 24.07% of preservative-free hospital ophthalmic preparations used in Rajavithi hospital were contaminated. The most commonly detected microorganisms were fungus, and molds (filamentous fungi; Aspergillus, Fusarium) were predominant followed by a wide range of Gram-positive bacteria. The statically significant factor related to number of eye drops used per person. Because contamination may originate from human or environmental flora, it is important to instruct patients about installation techniques and how eye drops should be stored. Medical and nursing staff should take precautions when administering and handling the ophthalmic preparations in order to minimize the risk of microbial contamination.

## Study Approval

The authors confirm that any aspect of the work covered in this manuscript that involved human patients was conducted with the ethical approval of all relevant bodies and the study was performed in accordance with the Declaration of Helsinki, and the protocol was approved by the Ethics Committee of Rajavithi Hospital, Thailand (approval number:78/2562).

## Author Contributions

The authors confirm contribution to the paper as follows: Conception and design of study: SC; Data collection: PH; Analysis and interpretation of results: SC, PH; Drafting the manuscript: SC, PH, AG, PR. All authors reviewed the results and approved the final version of the manuscript.

## Acknowledgments

We wish to thank various people in Rajavithi Hospital for their contribution to this project: nurses and ophthalmologists from the Inpatient Department for collecting and handling specimens, staff in Research Department for their valuable technical support, and staff in Microbiology Department for their help in data recording and analysis, and identification of microorganisms from the cultured specimens.

## Funding

This work was supported by Research funds of Rajavithi Hospital, Thailand.

## Conflict of Interest

The authors declare that they have no known competing financial interests or personal relationships that could have appeared to influence the work reported in this paper.

## Abbreviations

BUDs: Beyond-use dates
USP: United States Pharmacopeial Convention
CSPs: Pharmacy compounded sterile preparations
VA: VAvisual acuity
CoNS: coagulase-negative staphylococci
MALDI-TOF MS: Matrix-assisted laser desorption ionization-time of flight mass spectrometry
PMF: peptide mass fingerprint
